# A Shrewd Calculation on the Profits of Druggists

**Published:** 1880-05

**Authors:** 


### A SHREWD CALCULATION ON THE PROFITS OF DRUGGISTS.

ONE of the leading newspapers of the State, in discussing the question of percentages paid by druggists, says of a case in point: "If the actual cost of drugs used in the prescription were known, it would probably appear that fifty cents would have covered everything, and that all the rest was clear profit—or sheer plunder." Now, we suppose the cost of setting up an advertisement of three lines in that newspaper would amount to about three cents, and yet one would have to pay a dollar for it. Would the editor regard the ninety-seven cents as sheer plunder? Newspaper men had better not talk about profits of apothecaries.—*Pacific Med. and Surg. Journal.*

